# Effect of Composition and Thickness on the Perpendicular Magnetic Anisotropy of (Co/Pd) Multilayers

**DOI:** 10.3390/s17122743

**Published:** 2017-11-28

**Authors:** Bharati Tudu, Kun Tian, Ashutosh Tiwari

**Affiliations:** 1 Department of Materials Science and Engineering, University of Utah, Salt Lake City, UT 84112, USA; bharati.tudu@jadavpuruniversity.in (B.T.); cindy.tian@utah.edu (K.T.); 2 Department of Physics, Jadavpur University, Kolkata-700032, India

**Keywords:** perpendicular magnetic anisotropy, sputtering, multilayers, magnetic tunnel junctions

## Abstract

Magnetic materials with perpendicular magnetic anisotropy (PMA) have wide-ranging applications in magnetic recording and sensing devices. Multilayers comprised of ferromagnetic and non-magnetic metals (FM–NM) are interesting materials, as their magnetic anisotropy depends strongly on composition and growth parameters. In this context, (Co/Pd) multilayers have gained huge interest recently due to their robustness and tunable PMA. Here, we report a systematic study of the effect of composition on the magnetic anisotropy of (Co/Pd) multilayers grown by Direct Current (DC) magnetron sputtering. Four different series of (Co/Pd)_×10_ multilayers with different thicknesses of Co and Pd were examined. Vibrating sample magnetometery was used to determine the magnetic anisotropy of these films. X-ray diffraction and transmission electron microscopy experiments were performed to understand the structural morphology of the films. Our results showed that (Co/Pd)_×10_ multilayers exhibit PMA when the Co to Pd ratio is less than or equal to 1 and the thickness of Co layers is not more than 5 Å. Maximum effective anisotropy energy is shown by the films with a Co to Pd ratio of 1/3.

## 1. Introduction

Magnetic sensors have been applied to almost every sector of technology, as well as in our day-to-day life. In the last few years, sensors based on spintronics have gained particular attention due to their numerous advantages such as low power consumption, high sensitivity, compactness, and CMOS compatibility [1,2,3]. Materials with perpendicular magnetic anisotropy (PMA) are very interesting in this regard compared to in-plane anisotropy materials due to their ability to provide better storage density and thermal stability [4,5]. PMA magnetic media have already succeeded the in-plane media in hard disks [6]. In magnetoresistive random access memories (MRAM) also, PMA materials have performed better. With the discovery of the spin transfer torque (STT) phenomenon, a huge revolution has occurred in the MRAM industry [7,8,9]. Using the STT phenomenon, the magnetization state (‘0’ or ‘1’) of the free layer, which is used to store information in a magnetic tunnel junction (MTJ), can be reversed with a spin polarized current. This makes the MRAM device simpler, faster, low power consuming, and more efficient compared to the conventional field-induced MRAM. Till now, materials with in-plane anisotropy have been practically used in STT-MRAM [10]. However, PMA materials can provide superior STT-MRAM properties such as excellent thermal stability, lower power consumption, and increased storage density, which can take the MRAM technology below the 10 nm node [11].

Different categories of PMA materials have been studied for the perpendicular MTJ (p-MTJ), such as rare earth-transition metal films like GdFeCo or TbFeCo, *L*1_0_ ordered FePt or CoPt films, Heusler alloys such as Mn_3-δ_Ga, CoFeB alloys, and ferromagnetic/non-magnetic metal multilayers such as Co/Pt, Co/Pd, Co/Au, Fe/Pt, and Fe/Pd [4,5]. For their possible application in STT-MRAM, in addition to their high perpendicular anisotropy, these materials should also have high spin polarization, high tunneling magnetoresistance (TMR), low gilbert damping constant (α), and high thermal stability. In this context, Co-based multilayers are promising candidates due to their tunable PMA, spin-polarization, and α values [12,13,14]. In these multilayers, the sharp interfaces between the magnetic and non-magnetic lattices, as well as the strain in the magnetic layer, leads to the hybridization of the magnetic metal’s *3d* and non-magnetic metal’s *5d* orbitals, which thereby increases the perpendicular magnetic moment of the magnetic layer and gives rise to PMA [15,16,17]. With the increase in the number of sharp interfaces, the PMA gets stronger. For using such multilayer films in p-MTJ for STT-MRAM application, there are some specific requirements. These requirements are driven by the need for higher TMR, higher thermal stability (Δ), and a lower switching current (*I_c_*) needed for switching the magnetization of the free layer. High thermal stability demands large effective magnetic anisotropy energy, *K_eff_*, and a low switching current demands smaller values of the thickness, volume, magnetization, anisotropy field, and damping parameters of the free layer [4]. Also, the coercivity of the reference or fixed layer of the p-MTJ needs to be much higher than the free layer to ensure reliable preservation of data in the free layer against thermal fluctuation. Thus, proper knowledge of the Co-based multilayer system is needed for employing it as the reference layer and free layer in a p-MTJ. It is well established that Co/Pd multilayer can show PMA with Co film thickness ≤1 nm [15,16,17,18]. As the Co layer gets thicker than this, the system becomes magnetized in the in-plane direction dominated by the shape anisotropy [15]. However, not much studies have been done on the Co/Pd compositional dependence on the PMA.

In this study, we report a systematic investigation of magnetic anisotropy in Co/Pd multilayers deposited by Direct Current (DC) magnetron sputtering. Different series of Co/Pd multilayers were studied to understand the effect of the composition and thickness of the Co and Pd layers on the overall magnetic properties. The magnetic, structural, and compositional studies were performed using vibrating sample magnetometer (VSM), X-ray diffraction (XRD), high-resolution transmission electron microscopy (HR-TEM), and Energy-dispersive X-ray spectroscopy (EDS).

## 2. Materials and Methods

Four different series of (Co/Pd) multilayers with stack structure Pd_3_/(${\mathrm{Co}}_{t_{Co}}$/Pd_0.3 or 0.9_)_×10_/Pd_3_ with $t_{Co}$ values ranging from 0.1 to 0.8 nm and Pd_3_/(Co_0.2 or 0.4_/${\mathrm{Pd}}_{t_{Pd}}$)_×10_/Pd_3_ with $t_{Pd}$ values ranging from 0.1 to 1.8 were deposited on Si substrate by Direct Current (DC) magnetron sputtering at room temperature. The numbers in the subscript denote the thickness in nanometer. The 3 nm thick bottom Pd layer was used as a seed layer for (111) growth direction. The deposition chamber (Denton 635 Sputter system) was first evacuated to a base pressure of ~6 × 10^−7^ Torr. Depositions were carried out at a constant Ar pressure of 5 mTorr using Ar flow rate of ~35 sccm. Low deposition rates for both Co (0.093 Å/s) and Pd (0.151 Å/s) were used to ensure uniform growth of the films with sharper interfaces and less Co-Pd intermixing [19]. The deposition rate of Co was 0.093 Å/s and that of Pd was 0.151 Å/s, corresponding to a power of 10 W and 6 W, respectively, for all the samples. After the deposition, the magnetization hysteresis loops in the in-plane and out-of-plane magnetic fields were measured using a Microsense FCM-10 vibrating sample magnetometer (VSM). After subtracting the diamagnetic contribution from the substrate, the effective anisotropy, *K_eff_*, was calculated from the area enclosed between the easy and hard axis of the *M*-*H* curves [18]. The saturation magnetization per unit volume was calculated by dividing the magnetic moment by the total volume of the (Co/Pd)_×10_ multilayer [20]. The multilayer volume is obtained by multiplying the sample area with the total multilayer thickness. The error in the absolute values of the saturation magnetization was estimated to be approximately 10% and was mostly due to the uncertainty in determining the thicknesses of the layers. For structural characterization of the films, we performed XRD studies using a Bruker D2 Phaser X-ray Diffractometer with Cu-*Kα* radiation. A high-resolution JEOL 2800 S/TEM system was used for performing transmission electron microscopy (TEM) and energy dispersive spectroscopy (EDS) on selected samples.

## 3. Results and Discussion

### 3.1. Effect of Co Thickness

**Figure 1:**
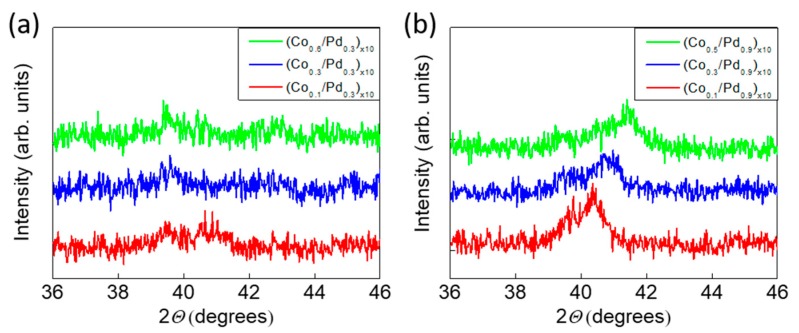
X-ray diffraction (XRD) (*θ*-2*θ*) patterns corresponding to samples of (**a**) series 1 and (**b**) series 2. A small peak at ~39.5° corresponds to the substrate.

To understand the effect of Co thickness on the magnetic properties of Co/Pd multilayers, two series of samples, one with thinner Pd layer [(${\mathrm{Co}}_{t_{Co}}$/Pd_0.3_)_×10_: series 1] and other with thicker Pd layer [(${\mathrm{Co}}_{t_{Co}}$/Pd_0.9_)_×10_: series 2], were prepared. The thickness of cobalt layer, $t_{Co}$, used was 0.1, 0.3, 0.4, 0.5, 0.6, and 0.8 nm. Figure 1a,b shows the XRD pattern of the films for the series 1 and 2. From the XRD data, it can be seen that for series 2, all the three samples show a peak around 40.4° corresponding to fcc (111) crystal orientation. The (111) peak around 40.4° shifts towards higher *θ* values with increasing Co thickness, which can be attributed to the lattice contraction of the (Co/Pd) system to match the lattice structure of the fcc Co layer. In contrast, no preferred crystal orientation was seen for the series 1 samples, which can be attributed to the thinner Pd layer. The thicker Pd layer facilitates the multilayer growth along (111) direction.

**Figure 2:**
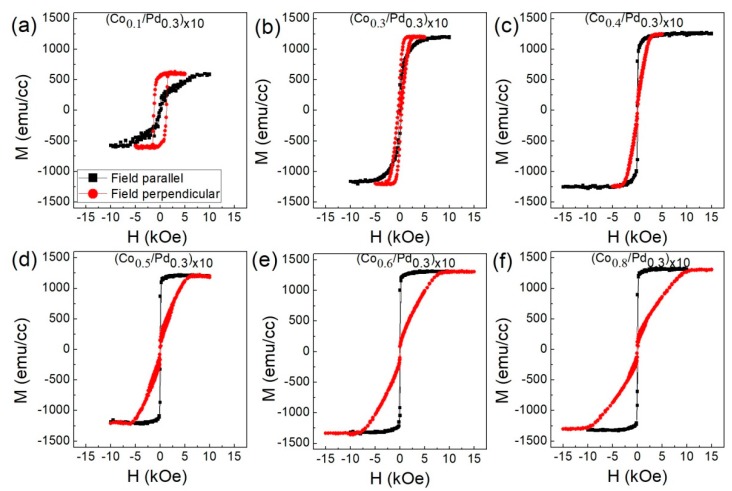
In-plane (black square) and out-of-plane (red circle) magnetic hysteresis loops for the sample (**a**) (Co_0.1_/Pd_0.3_)_×10_, (**b**) (Co_0.3_/Pd_0.3_)_×10_, (**c**) (Co_0.4_/Pd_0.3_)_×10_, (**d**) (Co_0.5_/Pd_0.3_)_×10_, (**e**) (Co_0.6_/Pd_0.3_)_×10_, (**f**) (Co_0.8_/Pd_0.3_)_×10_ of series 1.

**Figure 3:**
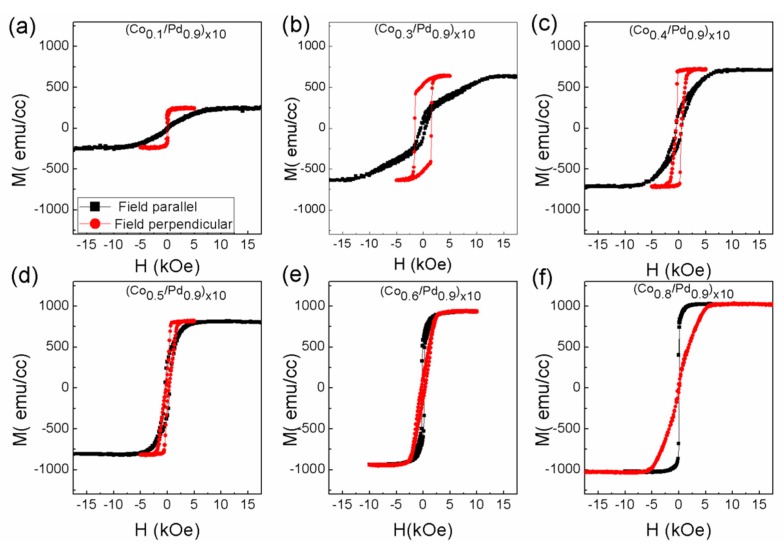
In-plane (black square) and out-of-plane (red circle) magnetic hysteresis loops for the sample (**a**) (Co_0.1_/Pd_0.9_)_×10_, (**b**) (Co_0.3_/Pd_0.9_)_×10_, (**c**) (Co_0.4_/Pd_0.9_)_×10_, (**d**) (Co_0.5_/Pd_0.9_)_×10_, (**e**) (Co_0.6_/Pd_0.9_)_×10_, (**f**) (Co_0.8_/Pd_0.9_)_×10_ of series 2.

The magnetization curves for these two series of samples measured along the in-plane and out-of-plane direction are shown in Figure 2 and Figure 3. The saturation magnetization of the films increases with the increase in the Co thickness, which is quite understandable, since the amount of Co per unit volume increases. The magnetization values of these samples are listed in Table 1.

**Figure 4:**
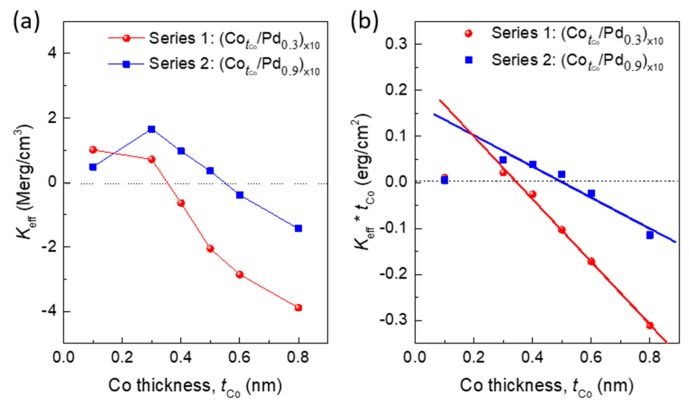
Plot of (**a**) effective anisotropy, *K_eff_*, of series 1 and series 2 as a function of $t_{Co}$; (**b**) *K_eff_* × $t_{Co}$ as a function of $t_{Co}$, along with the straight line fit; red colour represents series 1 and blue colour represents series 2.

Using the magnetization data, effective anisotropy energies (*K_eff_*) for all the samples were calculated. Figure 4a shows the plot of *K_eff_* with the thickness of Co layer. A positive *K_eff_* value describes a system with out-of-plane preferred direction of magnetization (or PMA), whereas a negative *K_eff_* value describes an in-plane magnetized system. It can be seen that PMA is obtained in some samples of both series 1 and series 2. Series 1 samples with thinner Pd layer (0.3 nm) lose PMA substantially with the increase of Co layer thickness, and PMA is obtained up to the Co thickness of 0.3 nm only. Series 2 samples with thicker Pd layer (0.9 nm) show comparatively higher PMA, and PMA is obtained up to the Co thickness of 0.5 nm. Here, the growth along (111) direction can be attributed to the enhanced PMA [21]. To understand the anisotropy behaviour clearly, the volume and interface anisotropies were calculated for these two series. The effective anisotropy energy has contribution from both the volume and interface, which follows the relation [18]:

(1) $$K_{eff}=K_{v}+2\frac{K_{s}}{t}$$

where *K_v_* is the volume anisotropy, *K_s_* the interface anisotropy, and *t* the thickness of the magnetic layer.

The interface anisotropy, which is mainly responsible for the PMA, can be found for a multilayer of particular Pd thickness by plotting the *K_eff_* × $t_{Co}$ as a function of $t_{Co}$ and calculating the intercept (=2*K_s_*) from the curve [18,19,20,21,22]. Figure 4b shows the plot of *K_eff_* × $t_{Co}$ with $t_{Co}$. for the two different series. It can be seen that for both the series, *K_eff_* × $t_{Co}$ deviates from its negative-slope linear behavior below the Co thickness of 0.3 nm and 0.4 nm, respectively. This effect could be due to the coherent (uniform lattice strain in the layer) and incoherent growth respectively below and above a critical magnetic layer thickness [23,24]. The interface anisotropy, *K_s_*, calculated from the intercept of the linear fit, is 0.117 erg/cm^2^ and 0.085 erg/cm^2^ for series 1 and series 2, respectively, which is comparable to the values reported for sputtered Co/Pd multilayers [15,16,17,18]. The volume anisotropy, −*K_v_*, is found to be 6.78 × 10^6^ erg/cm^3^ for series 1 and 3.37 × 10^6^ erg/cm^3^ for series 2. The comparatively lower volume anisotropy of series 2 samples helps the system to favor PMA up to a Co thickness of 0.5 nm. Beyond 0.5 nm, the system prefers in-plane anisotropy. On the other hand, for the series 1 samples, this critical thickness of Co is only 0.3 nm, beyond which the system prefers in-plane anisotropy.

### 3.2. Effect of Pd Thickness

**Figure 5:**
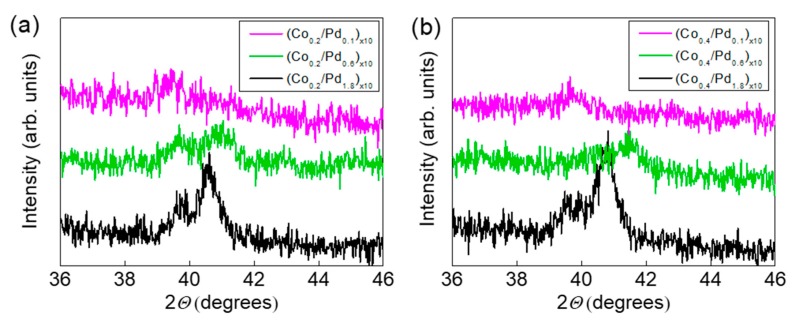
XRD (*θ*-2*θ*) patterns corresponding to samples of (**a**) series 3 and (**b**) series 4. A small peak at ~39.5° corresponds to the substrate.

To understand the effect of Pd thickness on the magnetic behavior of Co/Pd multilayers, two other series, one with Co layer of thickness 0.2 nm [(Co_0.2_/${\mathrm{Pd}}_{t_{Pd}}$)_×10_: (series 3)] and the other with Co layer thickness of 0.4 nm [(Co_0.4_/${\mathrm{Pd}}_{t_{Pd}}$)_×10_: (series 4)], were prepared. The thickness of palladium, $t_{Pd}$, used was 0.1, 0.3, 0.6, 0.9, and 1.8 nm. It is to be noted that we have not used Co layer thicker than 0.4 nm, since this is the average layer thickness (as found from the analysis of series 1 and 2), above which Co/Pd multilayer system prefers in-plane anisotropy. Figure 5a,b shows the XRD patterns of the films for series 3 and 4. In both the cases, no such peak along (111) direction is observed for the samples with a 0.1 nm thick Pd layer, which is obvious, but as the thickness of individual Pd layer increases, an enhancement in the intensity of (111) peak is observed.

**Figure 6:**
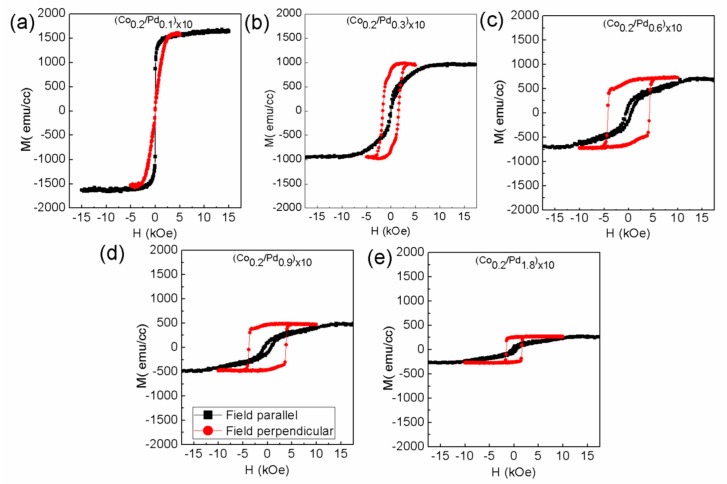
In-plane (black square) and out-of-plane (red circle) magnetic hysteresis loops of sample (**a**) (Co_0.2_/Pd_0.1_)_×10_, (**b**) (Co_0.2_/Pd_0.3_)_×10_, (**c**) (Co_0.2_/Pd_0.6_)_×10_, (**d**) (Co_0.2_/Pd_0.9_)_×10_, (**e**) (Co_0.2_/Pd_1.8_)_×10_ of series 3 with (Co_0.2_/${\mathrm{Pd}}_{t_{Pd}}$)_×10_.

**Figure 7:**
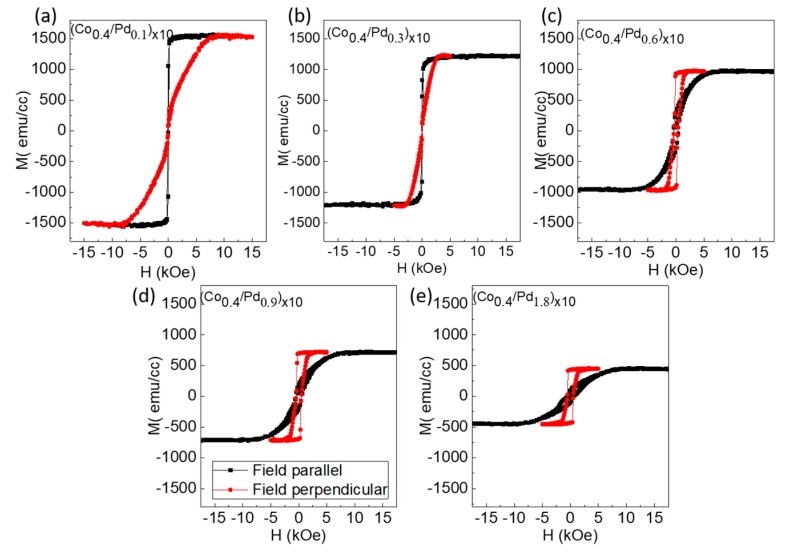
In-plane (black square) and out-of-plane (red circle) magnetic hysteresis loops of sample (**a**) (Co_0.4_/Pd_0.1_)_×10_, (**b**) (Co_0.4_/Pd_0.3_)_×10_, (**c**) (Co_0.4_/Pd_0.6_)_×10_, (**d**) (Co_0.4_/Pd_0.9_)_×10_, (**e**) (Co_0.4_/Pd_1.8_)_×10_ of series 4 with (Co_0.4_/${\mathrm{Pd}}_{t_{Pd}}$Pd)_×10_.

**Figure 8:**
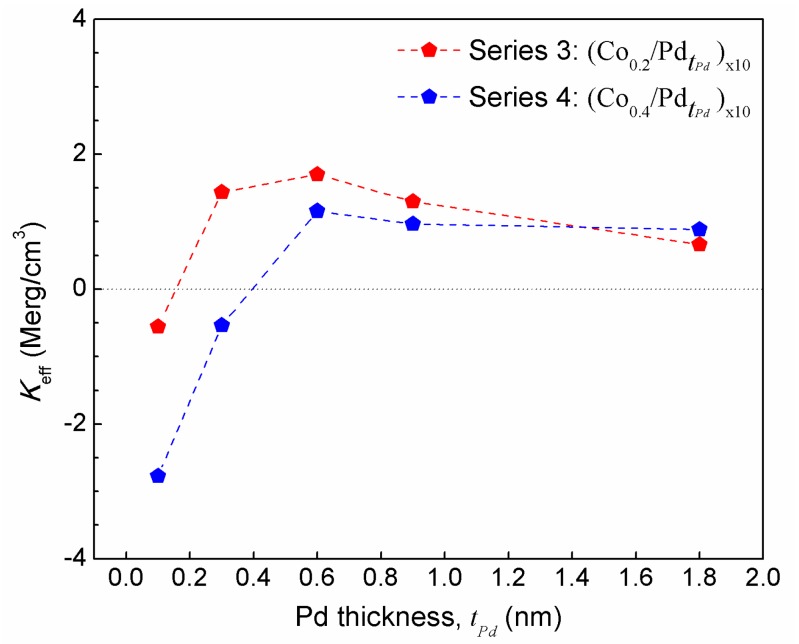
Effective anisotropy, *K_eff_*, as a function of $t_{Pd}$ for series 3 [(Co_0.2_/${\mathrm{Pd}}_{t_{Pd}}$)_×10_] and series 4 [(Co_0.4_/${\mathrm{Pd}}_{t_{Pd}}$Pd)_×10_] samples.

Figure 6 and Figure 7 show the *M*-*H* curves for the series 3 and 4 samples, measured in both in-plane and out-of-plane magnetic field. The saturation magnetization values for these samples are listed in Table 2. It can be seen that in both the cases, the 0.1 nm thick Pd layer is not sufficient to give rise to PMA. Series 3 starts to gain PMA when the Pd thickness from 0.3 nm onwards is increased. However, this thickness is not sufficient to bring PMA to series 4, which has thicker Co layers. It can be also seen that series 3 samples have different coercivity for different Pd thickness. First, the coercivity increases and then decreases with Pd thickness. This can be attributed to the non-monotonic change of inter-layer ferromagnetic coupling with the increase in spacer layer thickness, as reported earlier [25]. A maximum coercivity of 4.3 kOe is found for the sample (Co_0.2_/Pd_0.6_)_×10_. The squareness of the *M*-*H* curves is also maintained, showing a well-defined switching field. However, in case of series 4 samples, the typical squareness is not seen. Rather, the loop becomes narrow as the field is withdrawn to zero, indicating increased demagnetizing field of the sample. The effective anisotropy energy of these two series of samples is shown in Figure 8.

In this case, the compositions with thinner Co layer (series 2) show improved PMA. On comparing the XRD data (Figure 5a,b), it can be seen that the (111) peak corresponding to series 4 samples with thicker Co layer is more prominent, with enhanced peak intensity compared to that of series 3 samples with thinner Co layer of 0.2 nm. Thus, one can expect enhanced PMA in case of series 4 samples, since the (111) orientation provided by Pd layer is considered to favor the PMA [21]. The weaker (111) reflection in series 3 samples indicates that at this thickness of Co layer (0.2 nm), the Co and Pd atoms are intermixed across the interface, and thus the Co atoms gain lattice strain throughout the film. This is in line with our analysis of series 1 and series 2 samples, in which we stated that below a Co thickness of ~0.3 nm in case of series 1 (with *t_Pd_* = 0.3 nm) and 0.4 nm in case of series 2 samples (with *t_Pd_* = 0.9 nm), coherent growth takes place. Thus, the coherent growth or the Co-Pd alloy formation is the cause of enhanced PMA in series 3 samples.

### 3.3. TEM Characterization

**Figure 9:**
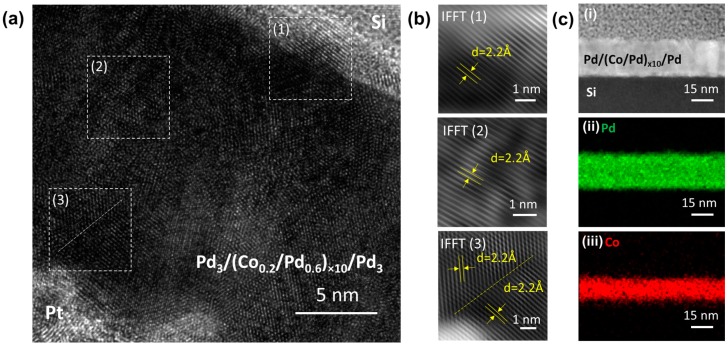
(**a**) High-resolution transmission electron microscopy (HR-TEM) image of sample (Co_0.2_/Pd_0.6_)_×10_ (cross-sectional view). (**b**) The inverse fast Fourier transform (IFFT) images show the crystalline lattices of areas (1), (2), and (3), which indicate the preferred crystal growth direction along (111) direction. Dashed line in area (3) indicates the grain boundary. (**c**) Scanning transmission electron microscopy image of the film cross-section (i) and corresponding energy-dispersive X-ray spectroscopy (EDS) elemental mapping images showing the distribution of Pd (ii) and Co (iii) in the multilayer stack.

TEM characterization of the sample (Co_0.2_/Pd_0.6_)_×10_, which showed the maximum PMA, was performed using a JEOL 2800 S/TEM system. For preparing cross-sectional TEM specimen, a 2-micron thick platinum layer was deposited above the multilayer, and the cross-section of the sample was obtained by cutting the sample using focused ion beam technique (FIB), as discussed in supporting information of Reference [26]. The cross-sectional TEM images of the sample were obtained at an acceleration voltage of 200 kV. Figure 9a shows a high-resolution cross-sectional TEM image from the (Co_0.2_/Pd_0.6_)_×10_ sample. In Figure 9b, we have shown the inverse Fast Fourier Transform (IFFT) images from three different regions of the film. In regions 1 and 2, we observed an almost single-crystal material, with a constant out-of-plane crystal lattice spacing ‘*d*’ of ~2.2 Å corresponding to the (111) growth direction. However, in region 3, we observed a grain boundary indicating textured nature of the films. In Figure 9c, we have shown the EDS elemental mapping of the (Co/Pd)_×10_ stack, which suggests a continuous distribution of Co and Pd across the entire multilayer.

### 3.4. Correlation Between Magnetic Anisotropy, Composition, and Lattice Parameters

**Figure 10:**
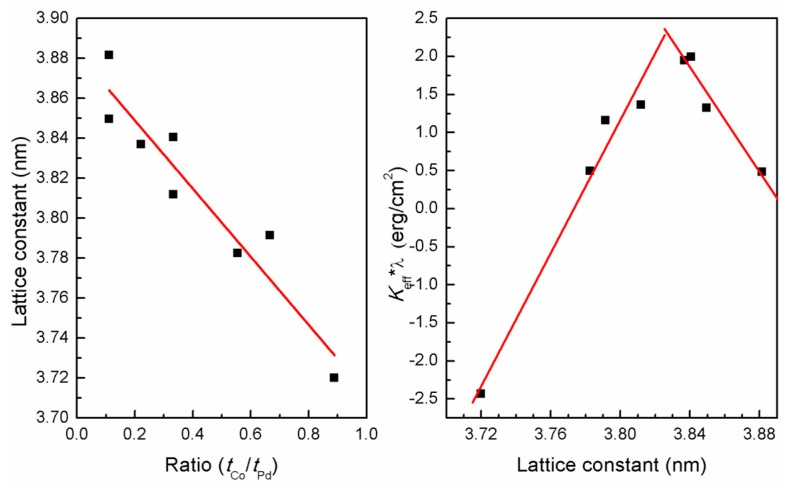
Plot of (**a**) lattice constant corresponding to the fcc (111) Co/Pd multilayers as a function of Co to Pd thickness ratio, and (**b**) effective anisotropy per bilayer (*t_Co_* + *t_Pd_*) of all the sample series as a function of the corresponding lattice constants.

To understand the relationship between anisotropy and composition better, we calculated the lattice parameter for all the samples showing distinct fcc (111) growth direction from the XRD data. We found that the Co/Pd composition affects the lattice constants of the multilayer. As the fraction of Co increases (or decreases), lattice contracts (or expands) to match the lattice constant of fcc Co ~0.357 nm. Figure 10a shows the plot of lattice constant with the ratio of Co and Pd, in which it can be seen that the lattice constant decreases linearly with the increase in Co fraction. This result is consistent with earlier reports [27]. We calculated the effective anisotropy per bilayer thickness, λ, and plotted it against the lattice constant, as shown in Figure 10b. We found that the effective energy per bilayer first increases linearly with the lattice constant. This observation is similar to the one reported on Co/Pd and Co/Pt multilayers by Ota et al. [28]. However, we found that the effective energy per bilayer starts to decrease linearly after a lattice constant value of ~0.383 nm. This can be attributed to the enhanced increase in the Pd fraction compared to the Co, which weakens the ferromagnetic coupling between the adjacent ultrathin Co layers.

**Figure 11:**
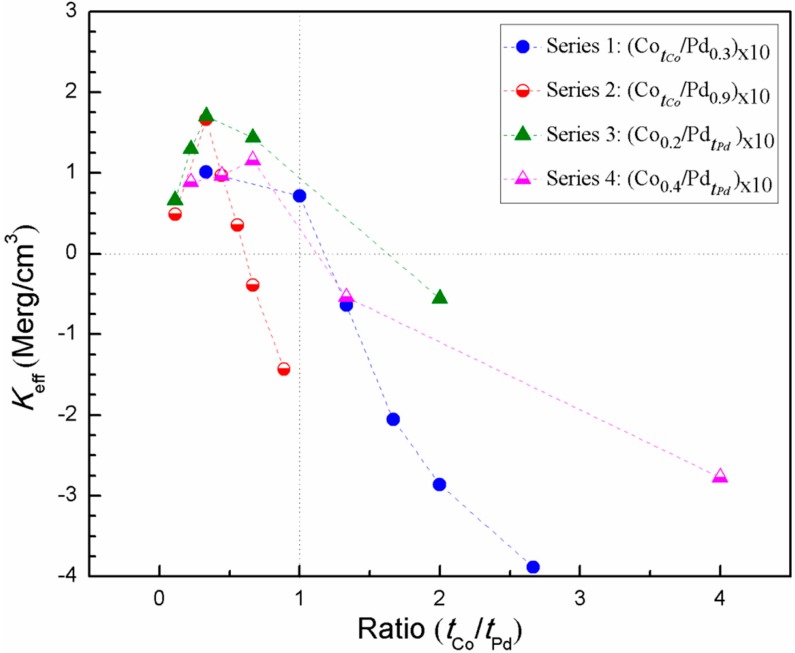
Plot of effective anisotropy of the entire sample series as a function of the ratio of Co and Pd thickness ($t_{Co}/t_{Pd}$).

Finally, we compared all the samples from series 1, 2, 3, and 4 to find a correlation between the effective anisotropy and its composition. Figure 11 shows the plot of effective anisotropy with the ratio of Co and Pd. In all the samples, the effective anisotropy first increases and then decreases with the Co:Pd ratio. We found that for all these four series, an out of plane magnetic anisotropy is observed for the ($t_{Co}/t_{Pd}$) ≤ 1. If the ratio is more than 1, the system attains in-plane anisotropy. All the series follow the trend of *K_eff_* > 0, for ($t_{Co}/t_{Pd}$) ≤ 1, with the exception of two samples from series 2, i.e., (${\mathrm{Co}}_{t_{Co}}$/Pd_0.9_)_×10_ with *t_Co_* = 0.6 nm and 0.8 nm, which is evident, since the in-plane volume anisotropy becomes dominant at this Co thickness range. The maximum perpendicular magnetic anisotropy is observed for the sample (Co_0.2_/Pd_0.6_)_×10_ (from series 3) with a value of 1.70 Merg/cm^3^, followed by sample (Co_0.3_/Pd_0.9_)_×10_ (from series 2) with a value of 1.66 Merg/cm^3^. Interestingly, in three series, 1, 2, and 3, the maximum PMA is found for the ($t_{Co}/t_{Pd}$) ratio of 1:3. However, for series 4, maximum perpendicular anisotropy is observed for the sample (Co_0.4_/Pd_0.6_)_×10_ with ($t_{Co}/t_{Pd}$) ratio of 2:3. This could be due to the shape anisotropy, which starts to dominate with thicker Co layer. In addition, with the increase in Pd layer thickness, the ferromagnetic interlayer coupling between individual Co layers start to weaken [25]. Thus, for this series, the optimum Co:Pd ratio for maximum PMA is found to be 2:3.

## 4. Conclusions

In conclusion, we have reported a systematic study of the magnetic anisotropy of the Co/Pd multilayers as a function of the different Co and Pd thicknesses. We found that for any composition, the Co:Pd thickness ratio plays an important role in the magnetic anisotropy. Our experimental results suggest that a Co:Pd ratio of less than or equal to 1 gives rise to PMA if Co thickness is not higher than 0.5 nm. An optimum Co:Pd ratio of 1:3 is found for the maximum PMA for thinner Co layers (0.1–0.3 nm), and a corresponding ratio of 2:3 is found for thicker Co layers (0.4 nm). A maximum PMA of 1.70 Merg/cm^3^ is found for the sample, with a Co thickness of 0.2 nm and a Pd thickness of 0.6 nm. From our experimental study, we conclude that a Co:Pd thickness ratio of 1:3 in the (Co/Pd)_×10_ multilayer system gives a higher perpendicular magnetic anisotropy with a square *M*-*H* hysteresis loops. This type of system can be used in magnetoresistance-based sensors, such as magnetic tunnel junctions, for various sensing applications.

## Figures and Tables

**Table 1:** Magnetization values of series 1 [(${\mathrm{Co}}_{t_{Co}}$/Pd_0.3_)_×10_] and series 2 [(${\mathrm{Co}}_{t_{Co}}$/Pd_0.9_)_×10_] samples corresponding to different values of $t_{Co}$.

Serial No.	Co Thickness, $t_{\mathrm{Co}}$ (nm)	Saturation Magnetization for Series 1 (emu/cm^3^)	Saturation Magnetization for Series 2 (emu/cm^3^)
1	0.1	350	140
2	0.3	700	350
3	0.4	800	431
4	0.5	875	500
5	0.6	933	560
6	0.8	1018	659

**Table 2:** Magnetization values of series 3 [(Co_0.2_/${\mathrm{Pd}}_{t_{Pd}}$)_×10_] and series 4 [(Co_0.4_/${\mathrm{Pd}}_{t_{Pd}}$)_×10_] samples corresponding to different values of $t_{Pd}$.

Serial No.	Pd Thickness, $t_{Pd}$ (nm)	Saturation Magnetization for Series 3 (emu/cm^3^)	Saturation Magnetization for Series 4 (emu/cm^3^)
1	0.1	933	1120
2	0.3	560	800
3	0.6	350	560
4	0.9	254	431
5	1.8	140	255
